# A spatiotemporal atlas of the lepidopteran pest *Helicoverpa armigera* midgut provides insights into nutrient processing and pH regulation

**DOI:** 10.1186/s12864-021-08274-x

**Published:** 2022-01-24

**Authors:** Panagiotis Ioannidis, Benjamin Buer, Aris Ilias, Sofia Kaforou, Michalis Aivaliotis, Georgia Orfanoudaki, Vassilis Douris, Sven Geibel, John Vontas, Shane Denecke

**Affiliations:** 1 Institute of Molecular Biology & Biotechnology, Foundation for Research & Technology Hellas, 100 N. Plastira Street, 700 13 Heraklion, Crete Greece; 2 Bayer AG, CropScience Division, R&D Small Molecules, 40789 Monheim, Germany; 3 Laboratory of Biological Chemistry, School of Medicine, Faculty of Health Sciences, Aristotle University of Thessaloniki, Thessaloniki, Greece; 4 Functional Proteomics and Systems Biology (FunPATh), Center for Interdisciplinary Research and Innovation (CIRI-AUTH), Balkan Center, Thessaloniki, Greece; 5 Department of Biological Applications and Technology, University of Ioannina, 45110 Ioannina, Greece; 6 Laboratory of Pesticide Science, Department of Crop Science, Agricultural University of Athens, Athens, Greece

**Keywords:** Lepidoptera, Expression atlas, Midgut, Helicoverpa

## Abstract

**Background:**

Caterpillars from the insect order Lepidoptera are some of the most widespread and destructive agricultural pests. Most of their impact is at the larval stage, where the midgut epithelium mediates the digestion and absorption of an astonishing amount of food. Although this tissue has been the subject of frequent investigation in Lepidoptera, a comprehensive expression atlas has yet to be generated.

**Results:**

Here, we perform RNA-sequencing and proteomics on the gut of the polyphagous pest *Helicoverpa armigera* across, life stages, diet types, and compartments of the anterior-posterior axis. A striking relationship between the structural homology and expression pattern of a group of sugar transporters was observed in the early larval stages. Further comparisons were made among the spatial compartments of the midgut, which suggested a putative role for vATPases and SLC9 transporters in the generation of alkaline conditions in the *H. armigera* midgut.

**Conclusions:**

This comprehensive resource will aid the scientific community in understanding lepidopteran gut physiology in unprecedented resolution. It is hoped that this study advances the understanding of the lepidopteran midgut and also facilitates functional work in this field.

**Supplementary Information:**

The online version contains supplementary material available at 10.1186/s12864-021-08274-x.

## Introduction

Agricultural pests damage human staple crops, contributing to global economic loss and food shortages. Particularly damaging are polyphagous caterpillars (Lepidoptera), which cause substantial crop damage across the world [1]. One of the most devastating of these pests is the cotton bollworm *Helicoverpa armigera*, which has a near global distribution and feeds on staple crops such as cotton, corn, and soybeans. *H. armigera* is known to undergo 5 larval molts (L1-L5) where the organism feeds intensely and rapidly increases its body mass several fold [2]. A deep understanding of the physiology of this species remains elusive on a molecular level.

As with all Lepidoptera, the midgut plays a key role in *H. armigera* larval physiology. This organ is a key interface between the outside world (lumen) and the body (hemocoel). Remarkably, the midgut is composed of only a single cell thick epithelium scaffolded on a basement membrane and visceral muscles [3]. Several cell types are present including the ubiquitous enterocytes (generalized midgut cells), enteroendocrine cells (enteropeptide secretors), goblet cells (thought to underpin midgut pH regulation), and stem cells that reside on the basement membrane and can replace the other cell types [4].

The physiological functions of the midgut are manifold [5], but primarily consist of selectively processing and absorbing nutrients while protecting the body from harmful elements of the environment. Nutrient absorption is thought to begin with secreted and membrane bound digestive enzymes such as trypsins and glycosidases that are ubiquitous in the midgut. The oligomers produced by these enzymes can then be absorbed either passively or via secondary active transporters into the body through enterocytes [6]. Running parallel to this is a network of proteins which prevent the penetration of toxic compounds such as plant secondary metabolites or pesticides. Drug metabolizing enzymes such as P450s have been shown to play a role in the midgut, and the role of transporters in this tissue is beginning to be explored [7, 8].

The lepidopteran midgut lumen is comprised of a highly alkaline pH. In some species this reaches upwards of pH 12, making it one of the most alkaline environments found in nature [9]. A series of studies in the 80s, 90s and 2000s demonstrated through electrophysiological techniques that this phenomenon is thought to be energetically driven by vacuolar ATPase (vATPase), which resides on the goblet cell apical membrane [9, 10]. Protons actively pumped out into the goblet cell cavity polarize the membrane and are subsequently re-absorbed by secondary 2H^+^/K^+^ transporters with the net effect of secreting potassium and stripping protons from the lumen [11, 12]. Carbonic acid or bicarbonate is also thought to be play a role in alkaline pH formation as it does in mosquitoes [13], through a combination of carbonic anhydrase and relevant ion exchangers transporters. Ultrastructural studies have further suggested that the anterior midgut is more metabolically active in this process [9]. Despite substantial biochemical evidence, little is known about the genes involved in this process in non-model insects [14].

Tissue expression atlases that report gene transcription across tissues or life stages have proven useful for researchers investigating molecular physiology in including model insects such as *Drosophila* and broader databases like Genevestigator [15, 16]. The falling cost of sequencing has also allowed this approach to be deployed in non-model organisms as was recently accomplished for the midgut of *Nezara viridula* [17]. The economic impact of Lepidoptera has generated substantial interest in these organisms and particularly the lepidopteran midgut since it is the target tissue of the widely used insecticidal *Bt* toxins. As a result, a multitude of midgut transcriptomes and proteomes (e.g. [18]) have been generated from Lepidopteran pests. However, there has been no systematic attempt to generate an atlas of gene expression in this tissue. Since it is often difficult to compare data across different studies, a more integrated view of midgut gene expression is not possible.

Here, we performed extensive RNA and protein sequencing across life stages, feeding conditions, and spatial compartments in order to provide an expression atlas of the *H. armigera* midgut. Comparisons suggested a wide variety of interesting physiological functions which were analyzed in more detail through annotation and phylogenetics. In the future, this comprehensive atlas will serve as a resource for researchers working on lepidoptera midguts and facilitate functional work in this field.

## Methods

### Insect rearing and tissue dissections

**Fig. 1:**
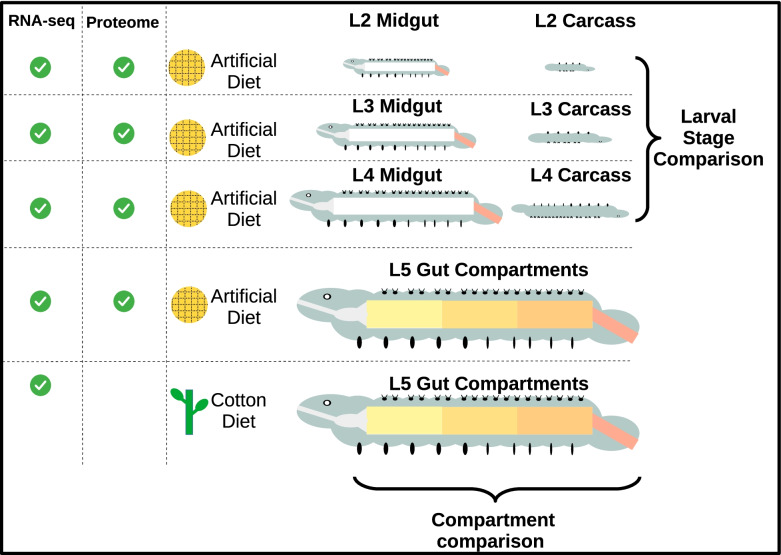
Schematic of gut sample collection: A collection of RNA-seq and proteome samples were taken from different larval stages and compartments of the *Helicoverpa armigera* alimentary canal. Collection of L2, L3, and L4 whole midguts allowed for the comparison across larval stages. Collection of different compartments of the L5 plant-fed midgut allowed for the comparison of how spatial regulation of gene expression looked across the midgut

A population of *H. armigera* was obtained from Serres, North Greece and maintained in the lab for several generations before this study. All individuals were reared at 24 ± 1 °C with a 16:8-h photoperiod on a standard Lepidoptera artificial diet based on corn flour (Table S1). Alternatively, individuals were raised from the L1 stage on cotton plants and further referred to as “plant-fed”. Larvae from the appropriate stage were dissected under RNAse free phosphate buffer saline (PBS). For the L2, L3, and L4 stages the midguts were dissected as a single unit, although the small size of the L2 larvae made it impossible to separate the minuscule foregut and hindgut. For the L5 larvae, guts were dissected out and separated into 5 compartments corresponding to the foregut (FG), anterior midgut (AMG), middle midgut (MMG), posterior midgut (PMG), and hindgut (HG). Four biological replicates from each gut condition were included in the analysis and each biological replicated consisted of at least five tissues (Fig. 1). All samples destined for RNA-sequencing were preserved in RNAlater and stored at − 80 °C until shipment. Tissue samples destined for proteomics were stored at − 80 °C in PBS.

### RNA-sequencing

RNA-sequencing was accomplished in collaboration with the McGill Genome Centre (Montreal, Canada). Total RNA was extracted from the above tissues using the QIAGEN RNA Extraction Mini Kit (QIAGEN, Germany), following the instructions of the manufacturer. cDNA was synthesized using the superscript III reverse transcription kit using 1 μg of RNA and poly dT primers. The Illumina TruSeq Library Prep Kit v2 (catalog number: #RS-122-2001) was used for generating the sequencing libraries, with IDT universal dual indices. Four replicates were sequenced for each sample. The raw sequencing reads generated in this paper are publicly available on NCBI (PRJNA716450).

### Proteomics

For L2, L3, and L4 midguts, dissected samples were sent to the Centre for Proteomics (Antwerp, Belgium) for gel-free analysis as was described previously [17]. For spatial compartment analysis of artificial diet fed L5 samples, a gel-based approach was used at the Proteomics Facility of the Institute of Molecular Biology and Biotechnology (Heraklion, Greece). This methodology split samples into a water-soluble and the membrane fraction and used bottom-up tandem mass spectrometry on a LTQ-Orbitrap XL coupled to an Easy nLC (Thermo Scientific) for proteome identification and relative quantification after protein fractionation on SDS-PAGE, as previously described [19]. For both approaches, a theoretical protein database was built using the gene set of the publicly available *H. armigera* genome (GCF_002156985.1 [20];). This database was subsequently used for identifying and relatively quantifying proteins from the mass spectrometry data using Proteome Discoverer 1.4.0 (Thermo Scientific) with Mascot 2.3.01 (Matrix Science) search algorithm and Scaffold (version 4.4.1.1, Proteome Software; Portland, OR [19];). The list of identified proteins was post-processed using custom scripts. While both approaches yielded either quantitative or semi-quantitative results, caution about overinterpreting these measurements led us to categorize proteins as “Present” or “Absent” in a sample based on whether the protein was detected in at least one replicate. For both methodologies,

### Transcriptomic analysis

The obtained raw sequences were first mapped on the publicly available *H. armigera* genome (GCF_002156985.1) using the HISAT2 short read aligner v2.1.0 [21] and the abundance for each of the predicted genes in the official gene set were calculated with featureCounts at the gene level [22]. For pairwise comparisons of the L2, L3, and L4 midgut samples to their corresponding carcass sample, EdgeR v3.30.3 [23] was used to find genes that were significantly (*q* < 1e-04) differentially expressed based on a false discovery rate correction. In order to see how gene expression varied across different compartments of the L5 midgut, fuzzy C-means clustering of genes was performed with the ‘Mfuzz’ R package v2.48.0. Cluster numbers were chosen after measuring minimum centroid distance using the “Dmin” function and an optimal fuzzifier parameter was estimated using the “mestimate” function. Only genes with a membership value (α) of > 0.7 were considered for plotting and further analysis.

### GO term functional enrichment analyses

For all gene sets gleaned from transcriptomic and proteomic comparisons, gene ontology (GO) term functional enrichment analysis was performed. GO terms for each gene were obtained from a previous publication reporting the original annotation of the *H. armigera* genome [20]. Fischer’s exact test was then used to test GO terms for enrichment based on their frequency in the specific gene group versus the entire genome using a custom R script (https://github.com/shanedenecke/Helicoverpa_gut_atlas). Significance values were corrected with the false discovery rate implemented through the *p.adjust* function in R. Terms with a false discovery rate below 0.001 were considered significant.

### Phylogenies

For selected gene families, phylogenetic trees were generated. The amino acid sequences of each family were aligned with MAFFT v7.450 using default parameters [24] and trimmed with Trimal v1.4 [25] using the “--automated1” algorithm. Trimmed alignments were used as inputs for a maximum likelihood tree using RAxML-NG v0.9.0 [26] with 500 bootstraps and the “LG + G8 + F” model. All trees and corresponding expression data were visualized with the ggtree package v2.2.4 in R [27].

### Identification of pH-related, detoxification, and protease genes

For the identification of genes relevant for pH regulation in the Lepidopteran midgut, we took two approaches. First, vATPase subunits and carbonic anhydrase enzymes were identified by a reciprocal best hits approach using the *D. melanogaster* sequences taken from the FlyBase *gene groups* “TYPE V P-ATPASES” and “CARBONIC ANHYDRASES” [28] as queries against the *H. armigera* predicted proteome. Pairs of genes that matched as best hits using the BLASTp e-value threshold of 1e-10 and query coverage threshold of 50% were included in the analysis. Second, Solute carrier (SLC) transporters from the *H. armigera* SLC9 family were predicted previously [29]. Because vATPase subunits and SLC9 transporters differed in terms of their absolute expression values by an order of magnitude, direct comparisons were accomplished by Z-score normalization using the mean expression of each gene across compartments.

For the identification of detoxification enzyme superfamilies including the cytochrome P450s (P450s), ATP-binding cassette transporters (ABCs), Carboxylesterases (CCEs), Glutathione-S Transferases (GSTs) and digestive enzymes (Trypsins and Chymotrypsins), we relied on the annotation from another study [20]. Because the ABC, P450, and GST superfamilies have only specific subgroups that have been associated with plant adaptation and detoxification, we chose to use only the CYP6 and CYP9 families from P450s, the ABCB, ABCC and ABCG families from ABC transporters, and the GSTE, GSTD, GSTS, and GSTT from the GST superfamily. For each tissue and each family, a two-sided *t*-test was used to see whether a gene was overexpressed in either the plant-fed or diet fed samples.

## Results and discussion

### Overview of the atlas

An expression atlas of the *H. armigera* larval gut was generated using 16 different spatio-temporal experimental conditions (Table S2; Fig. 1). Transcriptome sequencing was performed on whole midguts from artificial diet fed L2, L3, and L4 larvae along with their corresponding carcass samples. The midgut portion of these samples was also analyzed via proteomics, identifying a total of 2725 unique proteins in a gel-free approach. RNA-seq data was also generated for the L5 larval stage in five distinct gut compartments (Foregut (FG); Anterior midgut (AMG); Middle midgut (MMG); Posterior midgut (PMG); Hindgut (HG)) for both plant-fed and artificial diet-fed larvae. Complementary proteomics data was provided for artificial diet-fed larvae across the five L5 gut sections, and 3251 unique proteins were identified. In total this “Atlas” of expression data thus provides both transcriptomic and proteomic data on the 13,835 predicted *H. armigera* genes in the official gene set. Raw and summarized forms of this data are provided as a basis for future research on the lepidopteran midgut (Table S3, Table S4).

In order to check and sample replicability, we performed several principal component analyses of the transcriptomic data. First, RNA-seq data from the midguts of L2, L3, and L4 clustered independently from one another, but grouped very closely together compared to their corresponding carcass samples, which formed an independent cluster (Fig. S1). This indicates that the difference between midgut and carcass was far greater than any differences observed among life stages. Second, transcriptomic samples from L5 plant-fed midgut spatial sections clustered completely independently, suggesting large expression differences among compartments (Fig. S2). However, the artificial-fed transcriptomics generated from L5 gut sections showed substantial overlap between sections of the midgut along with overlap between the foregut and hindgut (Fig. S3). Lastly, comparisons of these individual artificial-fed sections with plant-fed sections revealed substantial differences due to dietary change (Fig. S4).

Given this overview of the data trends and quality, we thus chose to use the transcriptomic and proteomic data to focus on three different comparisons; (a) transcriptomic comparisons of midguts and carcass samples across different developmental stages (L2-L4), (b) proteomic and transcriptomic comparisons across the five different gut compartments of L5 larvae, and (c) transcriptomic comparisons between artificial diet-fed and plant-fed L5 compartments (Fig. 1; Table 1).

**Table 1:** Comparison overview: all comparisons made in the current study are shown in tabular format. Each column contains an experimental variable (Laval stage, Tissue compartments, Diet, Dataset) and the bold text represent which variable is being used for comparative purposes

Larval stage	Compartment(s)	Diet	Dataset	Comparison
L5	**5 gut secitons**	Artificial	Proteomics	Proteins specific to a single compartment
L5	5 gut secitons	**Artificial + Diet**	Transcriptomics	DE expression between plant and diet
L5	**5 gut sections**	Plant	Transcriptomics	Fuzzy c-Means among compartments
L2,L3,L4	**Midgut, Carcass**	Artificial	Transcriptomics	DE expression between midgut and carcass
**L2,L3,L4**	Midgut	Artificial	Proteomics	Common proteins

### Comparisons across larval stages suggest conserved regulation of related sugar transporters

**Fig. 2:**
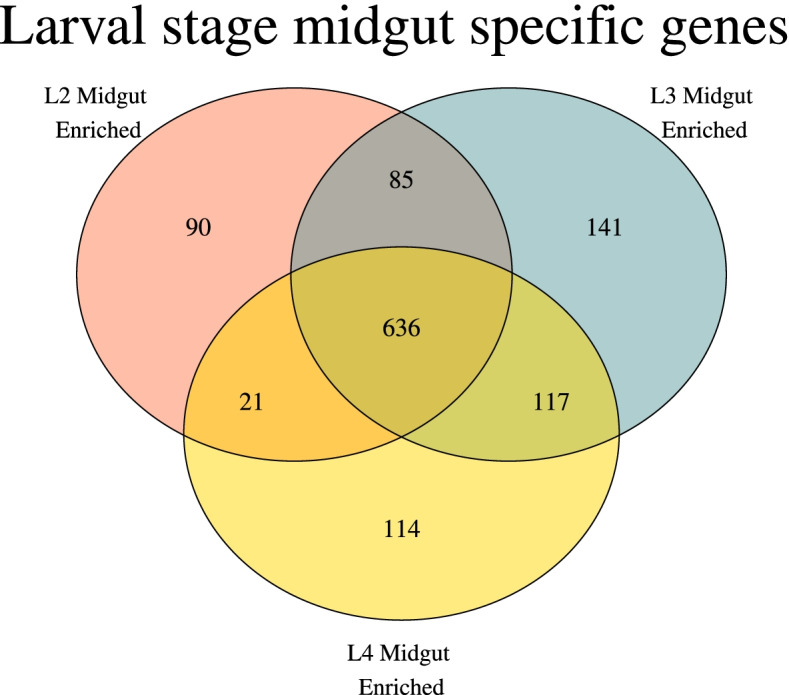
Midgut upregulation across larval stages: Genes that were upregulated in the midgut compared to the carcass for a particular larval stage were compared among each other. The majority of genes were midgut-upregulated in all larval stages, while a smaller percentage were only midgut-upregulated in a particular larval stage

The transcriptomic data corresponding to the midguts and carcasses from the L2, L3, and L4 larval stages were explored by focusing on genes overexpressed in the larval midgut compared to their corresponding carcass samples. Our analysis showed 833 upregulated genes in the gut tissue of L2 larvae, 980 genes in L3, and 889 genes in L4 (Fig. 2; Table S5). Almost half (636 out of the total 1205) gut overexpressed transcripts were commonly upregulated in all three comparisons, indicating a high degree of similarity across larval stages. GO term over-representation analysis (Table S6) showed that genes commonly overexpressed in the midgut of all larval stages are related to digestive functions such as lipid metabolism (GO:0006629) and proteolysis (GO:0006508). This finding was also observed in the stink bug *N. viridula*, and is commonplace among insects as it has been proven that they play an important role in the breakdown of proteins in the ingested food [17, 30, 31].

Genes upregulated in the midgut at only one specific larval stage were identified and further studied. In particular, we noticed that the GO term “transmembrane transport” (GO:0022857) was significantly enriched specifically at the L2 stage (*p* = 2 × 10^− 7^); this was explored further as a critical role for such nutrient transporters in the midgut had been previously suggested [6]. Cross referencing the transporters from this GO term with the recently published annotation of *H. armigera* transporters [29] suggested that organic ion transporters from the SLC22 family (*n* = 6) and sugar transporters from the SLC2 family (*n* = 3) contributed to this finding. These two families were thus explored separately (Table S7).

**Fig. 3:**
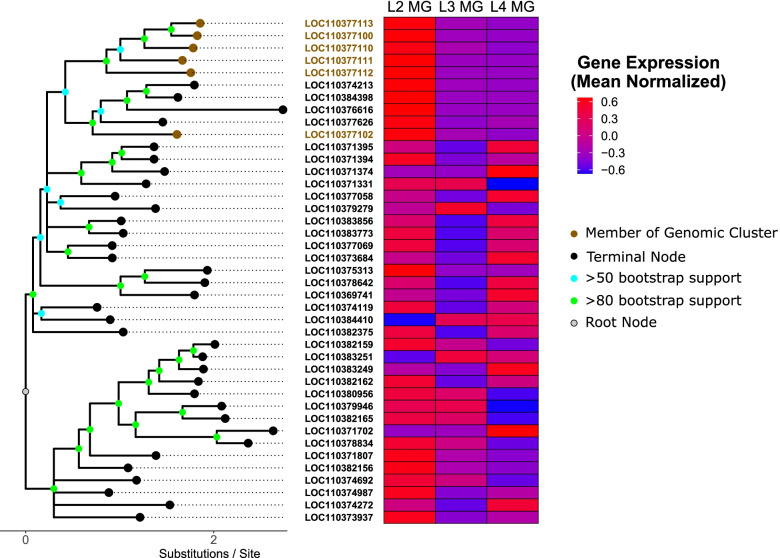
Comparison of homology with expression: Sequence homology and midgut expression was compared among genes from the SLC2 transporter family. (Left) A phylogenetic tree for all members of the tree with mean expression values over 1 TPM was made using RAxML-NG. Brown tip points signify presence on scaffold 139. The blue bracket signifies the group of related genes showing specific expression in the L2 midgut. (Right) The expression level of each SLC2 gene was plotted for the L2 (red), L3 (green), and L4 (blue) larval stages

Separate phylogenetic analysis of the SLC2 and SLC22 gene families were generated for genes above a minimum expression value (1 TPM) and juxtaposed against transcriptomic expression data from each larval stage. For the SLC2 family, there was a striking correlation between the protein homology indicated by the position on the phylogeny and gene expression indicated by the heat map (Fig. 3). A cluster of 10 related SLC2 genes showed consistent upregulation in the L2 midgut compared to other larval stages (Fig. 3). Six of these gene loci were found on the same scaffold (scaffold 139; NW_018395529). Four of these loci (LOC110377113, LOC110377100, LOC110377111, LOC110377112) were directly adjacent to one another, one was roughly 80 kb apart from this cluster and the remaining locus was about 300 kb from this cluster. The other 4 loci from this phylogenetic clade were dispersed among other scaffolds in the genome. In contrast, no relationship between expression and phylogenetic grouping was observed among the SLC22 transporters (Fig. S5).

These data suggest a relationship between the homology and expression pattern of SLC2 sugar transporters in the *H. armigera* midgut. The SLC2 family accounts for > 90% of the predicted transporters which act on dietary sugars and was recently shown to have undergone an expansion in Lepidoptera [29]. The cluster of six these transporters on scaffold 139 is suggestive of co-regulation observed commonly among adjacent genes. However, the other four related SLC2s showed a similar upregulation in the L2 midgut compared to other life stages, but were found at other genomic loci, suggesting the observed correlation was at least partially independent of chromosomal location. Previous studies have also considered the relationship between protein sequence similarity and transcript expression, finding similar expression patterns among orthologous genes from different species [32, 33]. However, it remains to be seen how widespread this phenomenon would be among insect transporters and functional work on these proteins is severely limited, especially in this large expansion of SLC2 proteins.

### Comparisons among L5 gut compartments suggests the basis of pH regulation

**Fig. 4:**
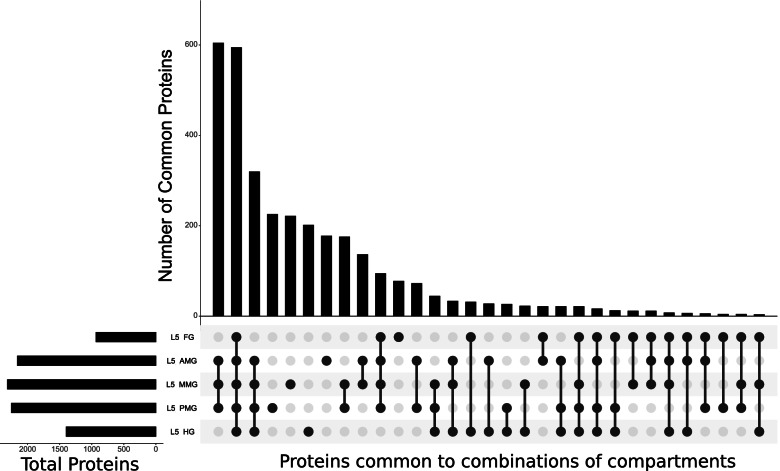
Proteomic spatial comparison: A comparison of all proteomics samples from different compartments of the artificial fed L5 midgut was accomplished with an “UpSet plot”. Total unique proteins identified in each sample are shown in the bottom left (Foregut: FG, Anterior midgut: AMG, Middle midgut: MMG, Posterior midgut: PMG, Hindgut: HG). The top portion of the figures shows the number of proteins common to each combination of tissues indicated by the grey dots in the bottom portion of the figure. The total proteins common to each combination of compartments (bottom center) is shown in quantitative terms (top center)

In order to understand spatial expression in the lepidopteran gut, we compared compartments along the *H. armigera* midgut. A proteomic comparison of all five compartments (FG, AMG, MMG, PMG, HG) of the alimentary canal of artificial diet-fed larvae suggested that the majority of proteins were found either exclusively in the three midgut compartments (AMG, MMG, PMG) or along the whole gut (Fig. 4; Table S8). GO term analysis of proteins specific to a single gut compartment only found significant enrichment of foregut genes in chitin binding, and “electron transfer activity” specific to the L4 proteome (Table S6).

**Fig. 5:**
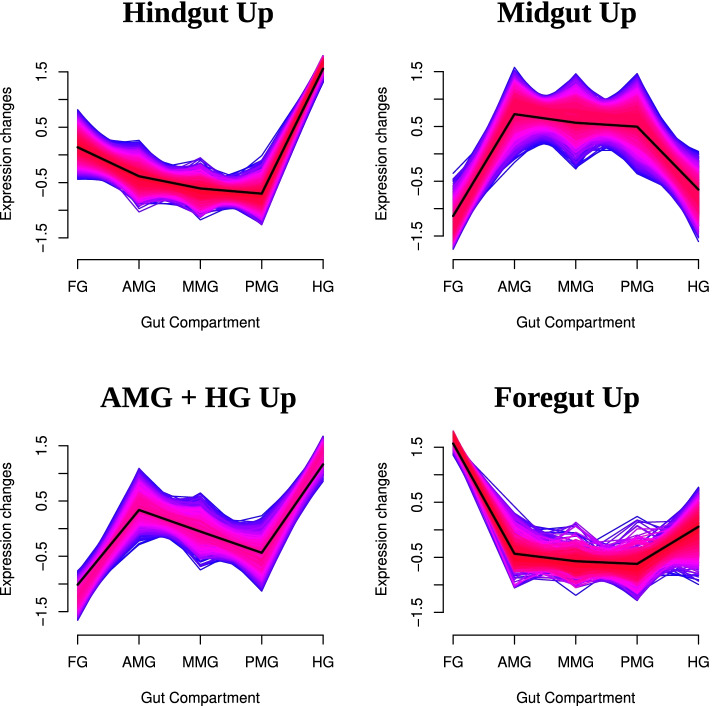
Fuzzy-C means clustering of spatial transcriptome: Transcripts were clustered into 4 groups using the RNA-seq data from the 5 plant-fed L5 midgut compartments and fuzzy-C means clustering. 3 of these clusters were notable in having one section of the alimentary canal upregulated (e.g., “Hindgut Up”), while the remaining cluster showed higher transcript abundance at the AMG and HG

Analysis of compartments was also achieved by considering transcriptomic data of the plant-fed midgut with fuzzy-c means clustering. Systematically testing the number of clusters as a function of minimum centroid distance found a significant drop-off after five clusters (Fig. S6). However, two of these clusters were very similar in terms of overall expression profile, so the number of chosen clusters was dropped to 4. This split genes into clusters which can generally be described as “midgut specific”, “hindgut specific”, “foregut specific” and an “AMG/HG” cluster which appeared to be lowly transcribed in the foregut, but very highly transcribed in the AMG and HG (Fig. 5; Table S9). Most interestingly, the AMG/HG cluster was enriched for translational machinery like “ribosome biogenesis” (GO:0042254) or “translation” (GO:0006412) along with “ATP hydrolysis coupled proton transport” (GO:0015991). Further exploration of the proton transport GO term revealed that the enrichment was caused almost entirely by vATPase subunits.

The identification of vATPases predominantly in the anterior midgut was noteworthy in the context of the current physiological understanding of the lepidopteran midgut pH gradient. The working model of pH alkalization in Lepidoptera involves the vATPase proton pump acting to generate electrical potentials across the goblet cell apical membrane, which then drives the absorption of protons into goblet cells via a 2H^+^/K^+^ exchanger [11]. However, goblet cells differ in their morphology along the length of the midgut. Anterior goblet cells have deeper cavities and microvilli filled with mitochondria compared to the posterior midgut which have shallower cavities and microvilli lacking mitochondria [9]. Therefore, the anterior midgut is thought to be the motive force behind pH gradients, which then return to near neutral pH in the hindgut.

**Fig. 6:**
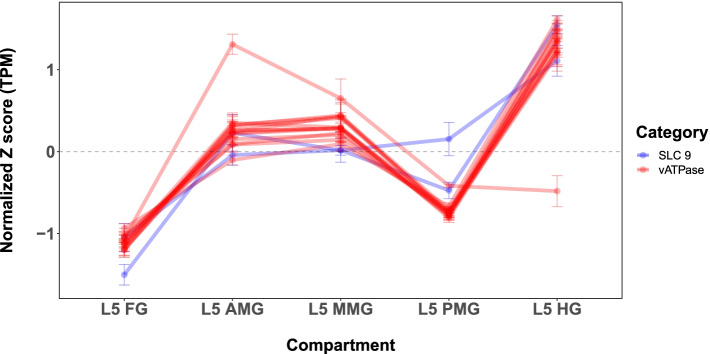
Co-expression of SLC9 and vATPase: Normalized Z-scores of 14 vATPase (red) and 2 SLC9 (blue) transcripts showing significant midgut expression were plotted along the axis of the midgut. The correlation between the red and blue lines represents the correlation between these different gene groups

Across eukaryotes, the only proteins known to handle 2H^+^/K^+^ ion stoichiometry are members of the SLC9 family (aka cation proton antiporters). Strikingly, two such SLC9 transporters (LOC110375477, LOC110370069) were identified within the same cluster as the vATPases and juxtaposition of their normalized expression values with vATPase subunits showed a strong correlation (Fig. 6; Table S10).

This co-regulation is in agreement with the previously suggested interaction of vATPase and potassium proton antiporters. Although this has long been hypothesized [34], so far the genetic evidence for SLC9 transporters has been severely limited. One recent study [35], examined related transporters in *D. melanogaster,* finding unusual stoichiometry capable of generating acid base transfer across epithelial tissues. Also interesting is the role of the hindgut, where pH rapidly returns to near neutral levels [9]. An increased vATPase and SLC9 presence in the hindgut is suggestive of ion movement, although this time with the goal of acidification rather than alkalization. As the vATPase complex appears to be able to increase or decrease pH depending on the epithelia under investigation [36], this hypothesis still fits with the transcriptomic data shown herein. Functional investigation of these phenomena in Lepidoptera will need to be undertaken in order to confirm or reject the role of these proteins in midgut alkalization.

### Comparisons among artificial and plant diets

Transcriptomic differences induced by diet were also investigated by comparing sections of the L5 midgut from larvae fed an artificial and plant (cotton) diets. GO term analysis of the differentially expressed genes between plant and artificial diet-fed individuals found many GO terms, but terms relating to oxidation such as “oxidation-reduction process” (GO:0055114) came up repeatedly in plant-fed samples (Table S6; Table S11). Furthermore, the term “proteolysis” was also upregulated in the plant samples. This was in line with previous studies, which found that monooxygenases such as cytochrome P450s (P450s) and digestive proteases such as trypsins were often upregulated in response to changes in diet [20]. We thus sought to expand upon these findings by comparing the expression of relevant subfamilies of detoxification enzymes such as P450s, ATP-binding cassette transporters (ABCs), Carboxylesterases (CCEs), Glutathione-S Transferases (GSTs) and digestive enzymes such as trypsins and chymotrypsins.

**Fig. 7:**
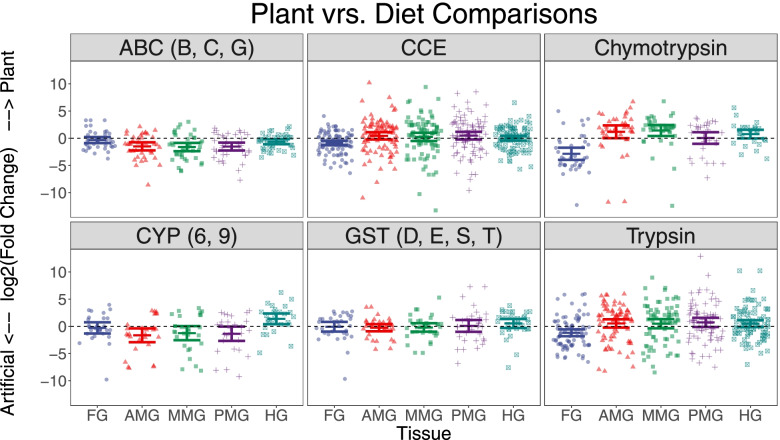
Effect of diet on detoxification and digestive gene families: Expression ratios for genes in subsets of relevant superfamilies were obtained by dividing the mean expression of a gut compartment in plant fed samples by its corresponding diet fed expression level. These values were then transformed using a Log_2_ transformations. Shapes and colors correspond to compartments while panels correspond to gene superfamilies. Black significance stars at the top of each dataset correspond to a student’s *t*-test *p*-value of <.01. Subsets of gene families were based on previous associations with detoxification and included “CYP6” and “CYP9” families from P450s, the “ABCB”, ABCC and ABCG families from ABC transporters and the GSTE, GSTD, GSTS, and GSTT GSTs

Relevant subsets of each gene family thought to be involved in detoxification (see Methods) were compared between plant based and artificial diet transcriptomic samples for each L5 compartment. ABC transporters were slightly but significantly downregulated on a plant-fed diet in four out of the five midgut compartments, while CCEs showed significantly higher expression in plant fed samples in the foregut and posterior midgut (Fig. 7; Table S12). No difference was seen in any compartment in the P450 or GST superfamilies. Both chymotrypsins and trypsins showed lower expression in the plant-fed foregut compared to foreguts reared on artificial diet, but this trend was reversed in some midgut sections (Fig. 7; Table S12).

Previous work has focused on the induction of such genes by different diets [37], but the response of individual genes in a family can vary substantially. For example, some members of the CYP6AE family are highly upregulated (4-50 fold) in the artificial diet fed samples depending on the section, while other members of the same family are overexpressed 4-9 fold in the plant-fed samples. Multiple members of this family have been implicated in the metabolism of plant secondary metabolites and they are localized to the same chromosomal location [38]. Similar results were found for trypsins and chymotrypsins, which showed individual members of each family being up to > 25 fold up or downregulated depending on the gene in question. A similar transcription pattern in trypsins and chymotrypsins was previously [31] found following feeding with protein inhibitors. While not a primary focus of this study, a more detailed functional investigation of these digestive enzymes would be useful in order to ascertain whether these changes are adaptive. In total, the variation of response within these gene families highlights the need to consider gene induction on a gene-by-gene basis.

## Conclusion

Here, we present a comprehensive atlas of the lepidopteran gut across different developmental stages and diets. The comprehensive sampling provided here in a single study should allow for more robust cross sample comparisons. For example, the upregulated transporter genes in the L2 stage (Fig. 3) would not have been identified by only considering one life stage. Additionally, the complementary transcriptomic and proteomic data allows for a more confident assignment of proteins to a given sample. Although we focused far more on the transcriptomic analysis for functional insights, cross-referencing with the herein generated proteomic data is available and can be used to exclude artifacts and gain additional understanding.

## Supplementary Information

**Additional file 1.**

**Additional file 2.**

**Additional file 3.**

**Additional file 4.**

**Additional file 5.**

**Additional file 6.**

**Additional file 7.**

**Additional file 8.**

**Additional file 9.**

**Additional file 10.**

**Additional file 11.**

**Additional file 12.**

**Additional file 13.**

**Additional file 14.**

**Additional file 15.**

**Additional file 16.**

**Additional file 17.**

**Additional file 18.**

## Data Availability

All sequencing reads generated in this study have been deposited on the sequence read archive (PRJNA716450; https://dataview.ncbi.nlm.nih.gov/object/PRJNA716450?reviewer=pav9p44j2mu2gne1epfag6kmoq). All other data is presented in supplementary files and raw data is available upon request.
