# Supplementary material for: A spatiotemporal atlas of the lepidopteran pest Helicoverpa armigera midgut provides insights into nutrient processing and pH regulation
Source: BMC Genomics. 2022 Jan 24;23:75. doi: 10.1186/s12864-021-08274-x (PMC8785469; doi:10.1186/s12864-021-08274-x)
Supplement: Supplementary file 1 — Additional file 1. [file 12864_2021_8274_MOESM1_ESM.pdf]

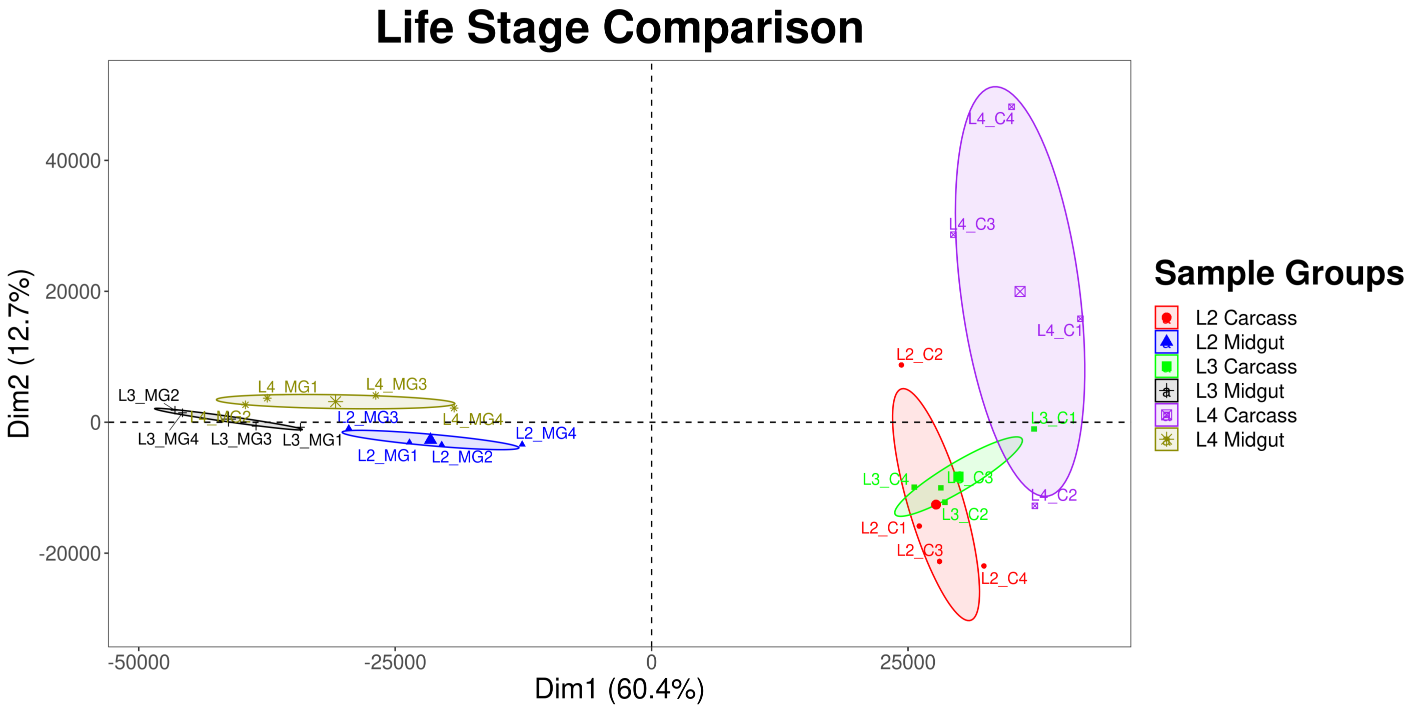

Figure S1: PCA plot of larval stage samples

The variation among replicates and samples of larval stage transcriptomes are shown. Colors and shapes correspond to different sample types. Ovals represents space occupied by each sample type. The X and Y axes signify the percentage of variation explained by each of the two principal components.
