# Supplementary material for: A spatiotemporal atlas of the lepidopteran pest Helicoverpa armigera midgut provides insights into nutrient processing and pH regulation
Source: BMC Genomics. 2022 Jan 24;23:75. doi: 10.1186/s12864-021-08274-x (PMC8785469; doi:10.1186/s12864-021-08274-x)
Supplement: Supplementary file 2 — Additional file 2. [file 12864_2021_8274_MOESM2_ESM.pdf]

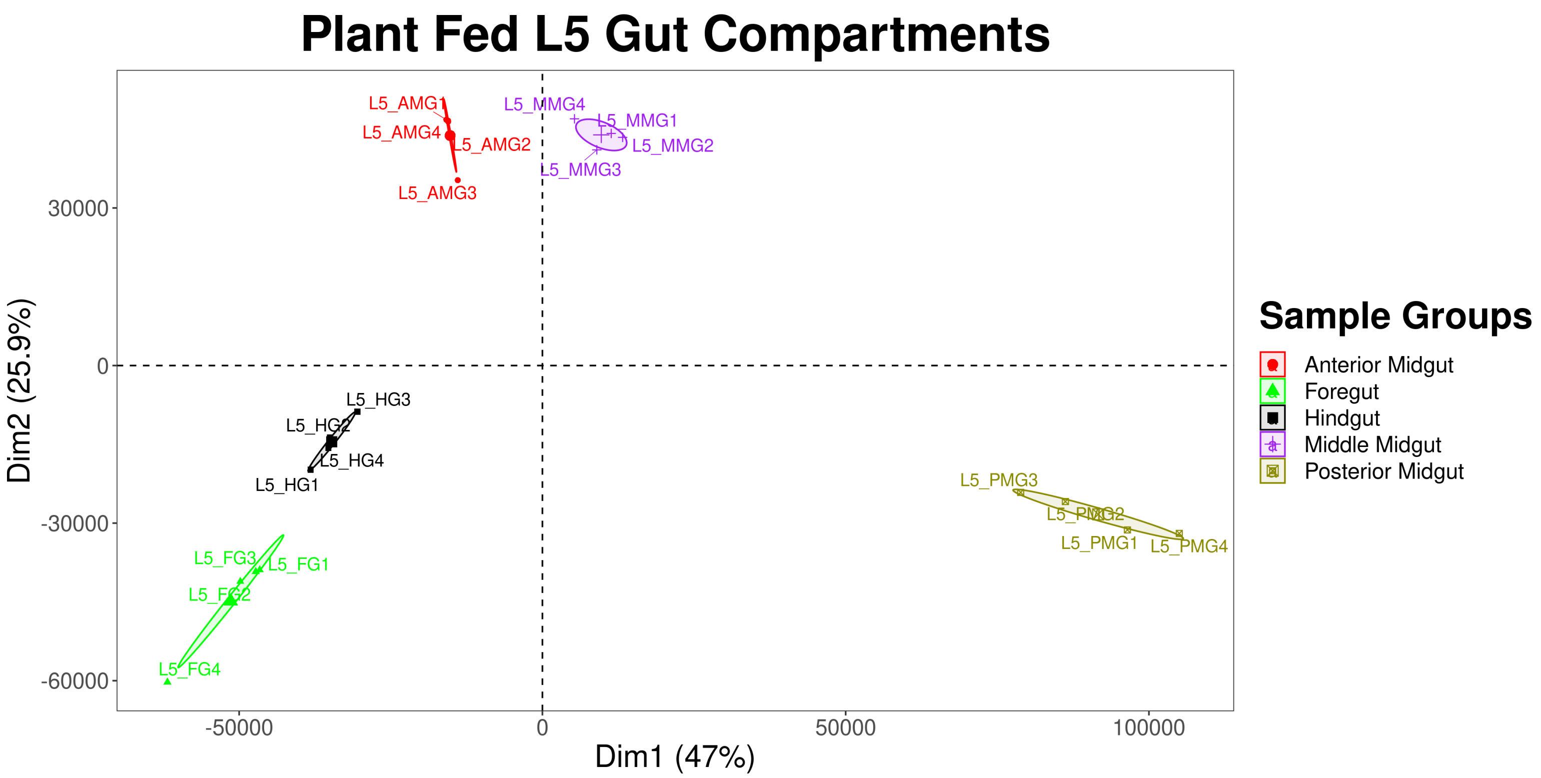

Figure S2: PCA plots of gut sections on plant-fed diet

The variation among replicates and of midgut sections on plant-based diet are shown. Colors and point shapes reflect different sample types (spatial sections of the midgut). Ovals represent the space occupied by each sample using the “ellipse.type = "confidence"” argument in the fviz_pca_ind function in R.
