# Supplementary material for: A spatiotemporal atlas of the lepidopteran pest Helicoverpa armigera midgut provides insights into nutrient processing and pH regulation
Source: BMC Genomics. 2022 Jan 24;23:75. doi: 10.1186/s12864-021-08274-x (PMC8785469; doi:10.1186/s12864-021-08274-x)
Supplement: Supplementary file 3 — Additional file 3. [file 12864_2021_8274_MOESM3_ESM.pdf]

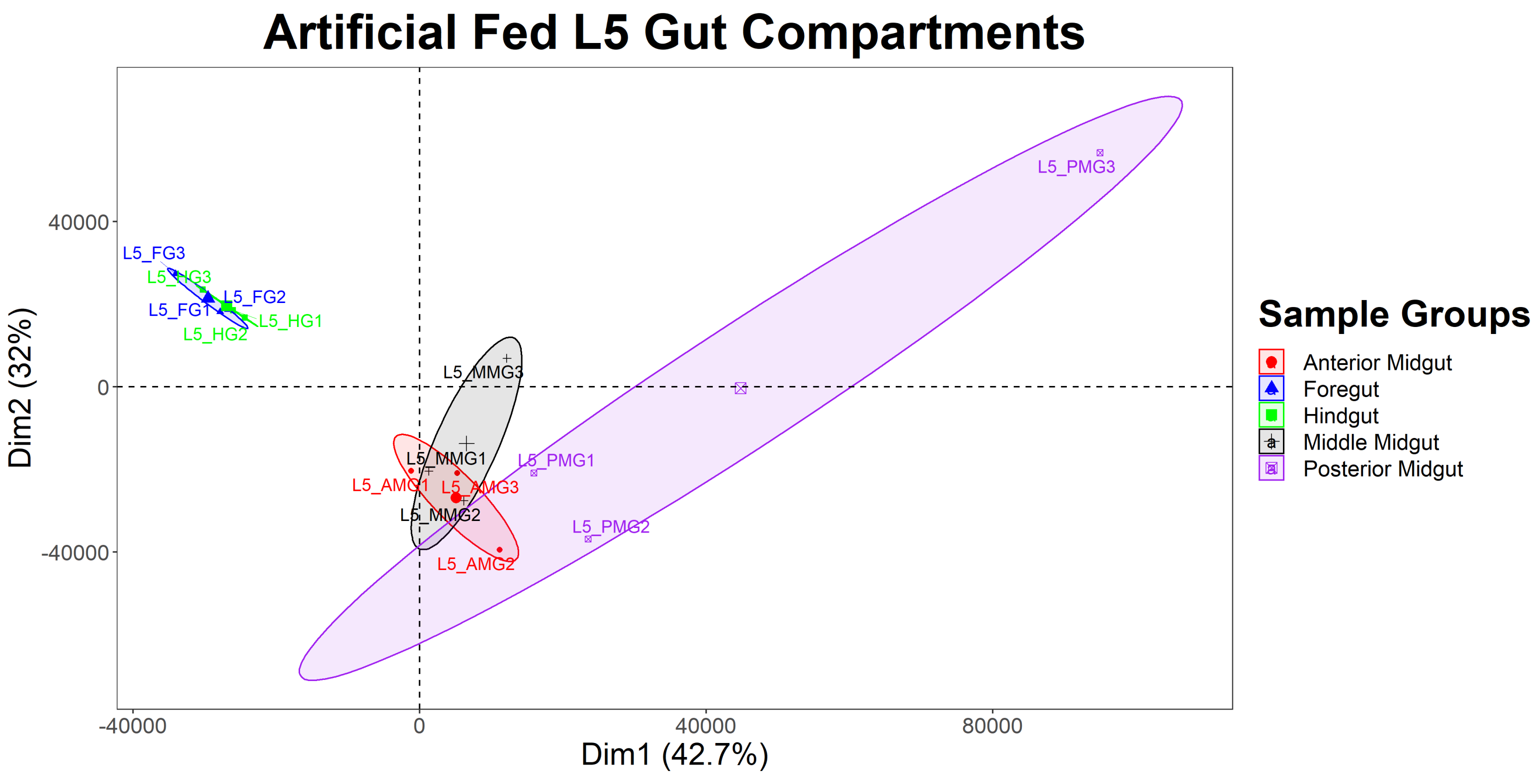

Figure S3: PCA plots of gut sections on plant-fed diet The variation among replicates and of midgut sections on artificial diet are shown. Colors and point shapes reflect different sample types (spatial sections of the midgut). Ovals represent the space occupied by each sample using the “ellipse.type = "confidence"” argument in the fviz_pca_ind function in R.
