# Supplementary material for: A spatiotemporal atlas of the lepidopteran pest Helicoverpa armigera midgut provides insights into nutrient processing and pH regulation
Source: BMC Genomics. 2022 Jan 24;23:75. doi: 10.1186/s12864-021-08274-x (PMC8785469; doi:10.1186/s12864-021-08274-x)
Supplement: Supplementary file 4 — Additional file 4. [file 12864_2021_8274_MOESM4_ESM.pdf]

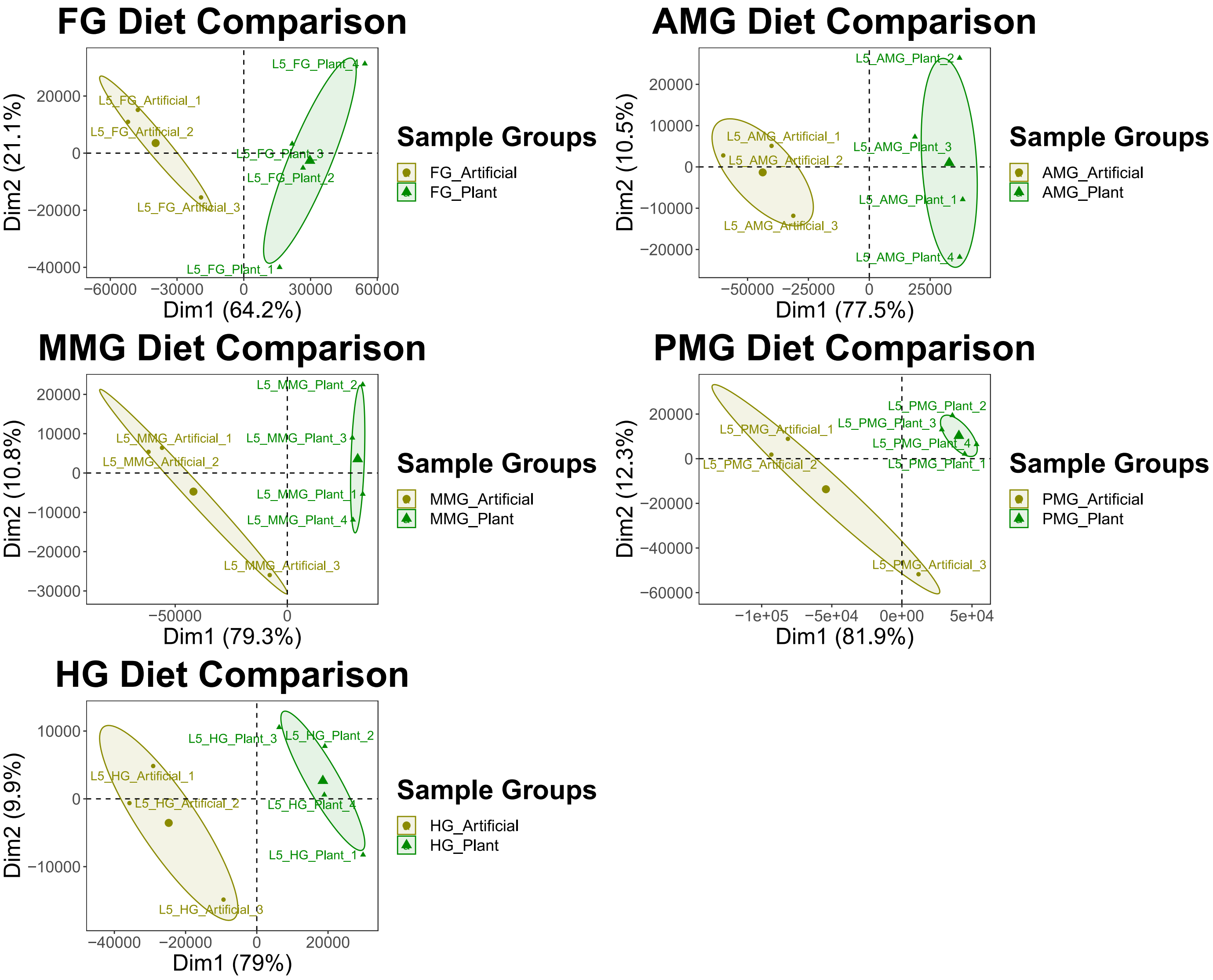

Figure S4: Diet PCA

The PCA plots reflecting the comparisons between the artificial and plant-based diets were made for each compartment. In each panel the green triangles correspond to plant-based samples, while the gold circles correspond to the artificial diet samples. An ellipse was drawn for each sample to suggest overall patterns with the “ellipse.type = "confidence" argument in the *fviz_pca_ind* function.
