# Supplementary material for: A spatiotemporal atlas of the lepidopteran pest Helicoverpa armigera midgut provides insights into nutrient processing and pH regulation
Source: BMC Genomics. 2022 Jan 24;23:75. doi: 10.1186/s12864-021-08274-x (PMC8785469; doi:10.1186/s12864-021-08274-x)
Supplement: Supplementary file 5 — Additional file 5. [file 12864_2021_8274_MOESM5_ESM.pdf]

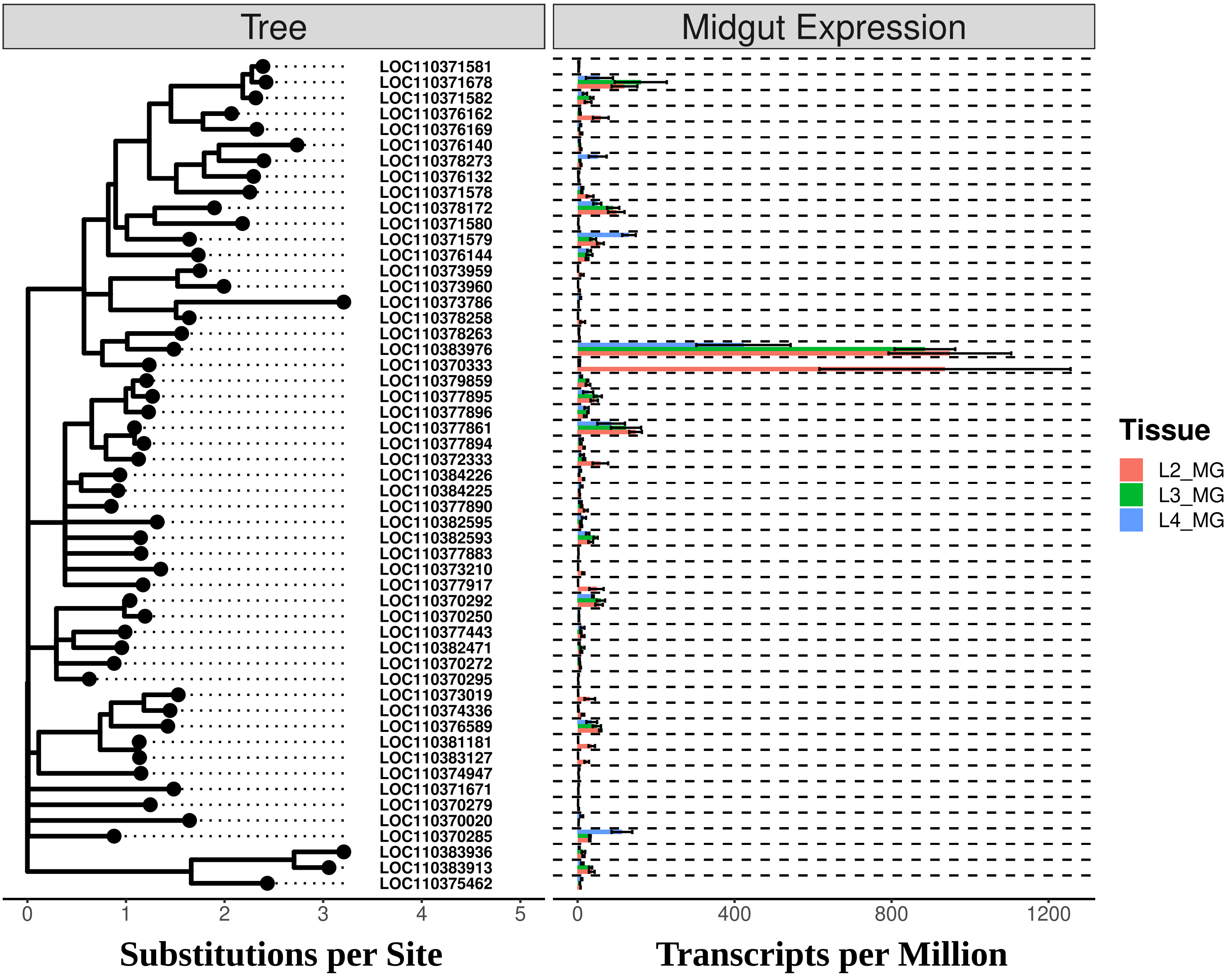


Figure S5: Comparison of homology with expression:

Sequence homology and midgut expression was compared among genes from the SLC22 transporter family. (Left) A phylogenetic tree for all members of the tree with mean expression values over 1 TPM was made using RAxML-NG. (Right) The expression level of each SLC22 gene was plotted for the L2 (red), L3 (green), and L4 (blue) life stages.
