# Supplementary material for: A spatiotemporal atlas of the lepidopteran pest Helicoverpa armigera midgut provides insights into nutrient processing and pH regulation
Source: BMC Genomics. 2022 Jan 24;23:75. doi: 10.1186/s12864-021-08274-x (PMC8785469; doi:10.1186/s12864-021-08274-x)
Supplement: Supplementary file 6 — Additional file 6. [file 12864_2021_8274_MOESM6_ESM.pdf]

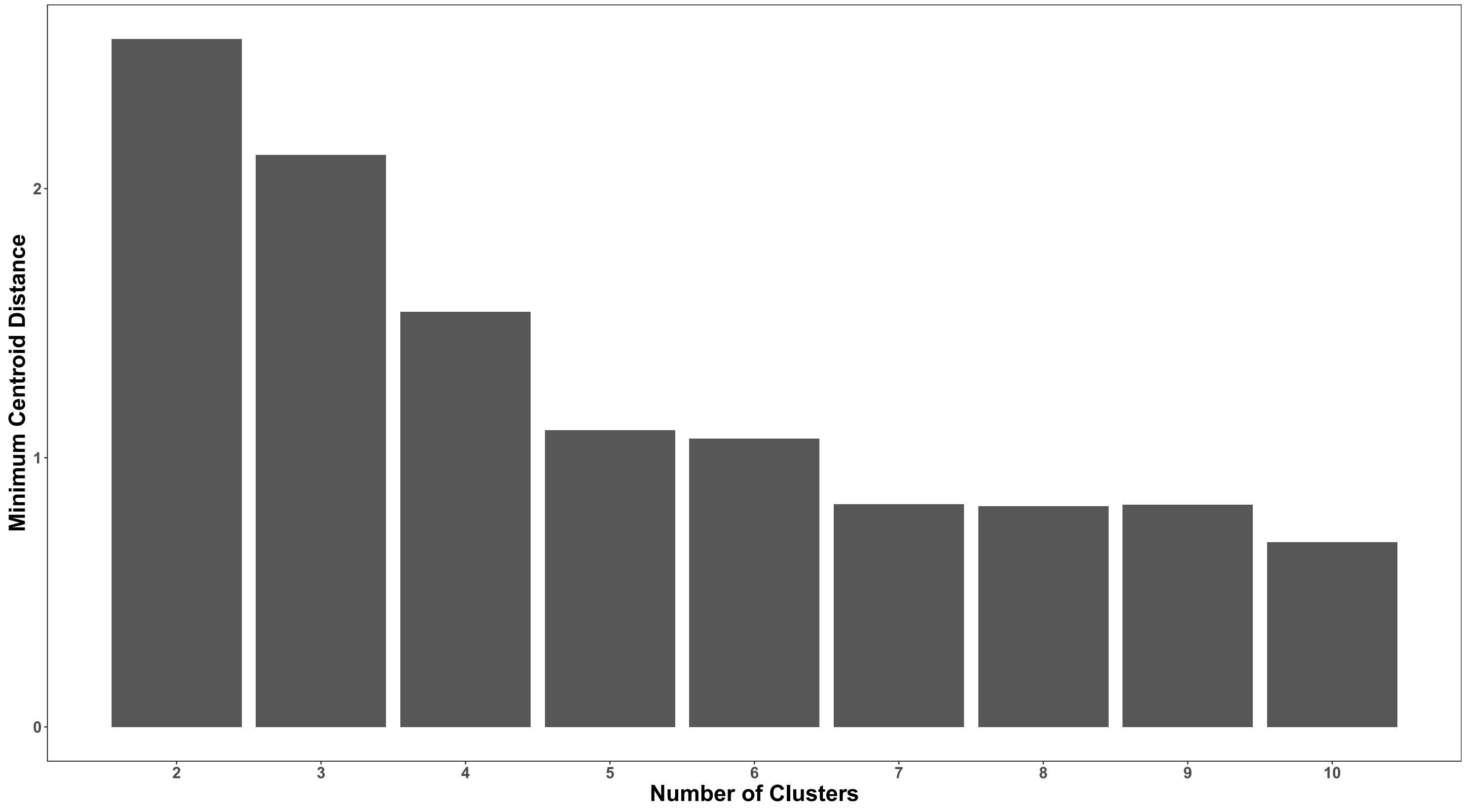

Figure S6: Fuzzy Clustering parameters

The minimum centroid distance of values for C-means clustering with various groupings is shown. On the y-axis shows the minimum centroid distance. The x-axis indicates the number of clusters in each run.
